# Recurrence of neovascular age-related macular degeneration after cessation of treat and extend regimen

**DOI:** 10.1038/s41598-022-19062-2

**Published:** 2022-08-30

**Authors:** Yuki Hirata, Akio Oishi, Yuki Maekawa, Eiko Tsuiki, Akira Machida, Junko Kurihara, Takashi Kitaoka

**Affiliations:** 1 Department of Ophthalmology and Visual Sciences, Graduate School of Biomedical Sciences, Nagasaki University, Sakamoto 1-7-1, Nagasaki, 852- 8102 Japan; 2 Department of Ophthalmology, Japanese Red Cross Nagasaki Genbaku Hospital, Mori-machi, Nagasaki, 852-8511 Japan

**Keywords:** Eye diseases, Macular degeneration

## Abstract

The appropriate timing of treatment cessation after treat and extend (TAE) regimen for age-related macular degeneration has not been established. This study aimed to investigate the incidence and risk factors of recurrence after cessation of the TAE regimen. We included patients who received and discontinued the TAE regimen, after extension of the treatment interval to ≥ 12 weeks. Forty-nine patients were included in the study. The estimated recurrence rates were 33% at 1 year and 48% at 2 years after treatment cessation, respectively. Good visual acuity at cessation and a large number of injections in the 6 months before cessation were significant risk factors. Higher chances of recurrence were associated with < 0.1 logarithm of the minimum angle of resolution (logMAR) at cessation (*P* < 0.002). Meanwhile, five patients with visual acuity ≥ 1.0 logMAR at cessation did not show recurrence. Among the 25 recurrences, two lines of vision loss were noted in only two cases after resumed treatment. This study confirmed the importance of the number of injections in reducing recurrence and the association between visual acuity and recurrence. Recurrence is generally well-controlled with resumed treatment.

## Introduction

Age-related macular degeneration (AMD) is the leading cause of blindness, particularly in developed countries^1^. The disease is classified into neovascular and atrophic forms, with the former characterized by macular neovascularization (MNV)^2^. MNV induces leakage, bleeding, and scarring, which subsequently impairs vision function.

The development of anti-vascular endothelial growth factor (VEGF) therapy has dramatically improved the visual outcome of neovascular AMD (nAMD)^3^. Anti-VEGF agents are administered in fixed, proactive, or reactive regimens. Among several treatment regimens, treat and extend (TAE) consists of injection and adjustment of the interval depending on the retinal exudative change^4^. The regimen is widely chosen due to its balanced cost and efficacy on visual function^5–7^. Meanwhile, anti-VEGF agents are expensive, and the treatment requires frequent injection and hospital visits, which are a major burden for elderly patients and the healthcare system^8,9^. It is impractical to continue anti-VEGF treatment endlessly in all patients. In fact, the dropout of patients due to socioeconomic or health reasons is not uncommon in clinical practice^10^. Prolonged anti-VEGF therapy may also be associated with a risk of endophthalmitis and macular atrophy^11,12^. Drug suspension, if possible, would decrease the burden on patients, the healthcare system, and the risk of complications.

Several reports have described cessation of the TAE regimen and the incidence of subsequent recurrences. In these reports, cessation of treatment was considered when there was no recurrence of exudative changes after the predetermined treatment interval (e.g., 12 or 16 weeks)^13–21^. However, the timing of treatment cessation may vary depending on the clinical course and the patient's preferences in real world clinical practice. The effect of the last treatment interval cannot be analyzed if all patients quit treatment with the same predetermined interval. To address this issue, we investigated real-world data to elucidate the incidence and risk factors of recurrence in patients with nAMD treated with the TAE regimen and discontinued treatment.

## Results

### Study population

In this study, 78 eyes were included, including 74 cases who met the inclusion criteria in the single eye and the right eyes of 4 bilateral cases. After excluding 26 eyes within the follow-up period of < 12 months and three eyes with other macular diseases (two diabetic macular edema and one macular hole), 49 eyes of 49 patients were included and analyzed.

### Baseline characteristics and treatment course of patients

Recurrence was observed in 25 patients (51%) at a median of 10.5 months (interquartile range [IQR] 8.1–18.2 months, range 4.4–36.5 months). The mean follow-up time was 34.8 ± 19.8 months (range 12.1–83.8 months). Sixty-four percent of the recurrences developed in the first year, and 20% were identified in the second year after cessation of treatment. Patient characteristics are shown in Table 1 and macular findings on OCT determined as recurrence after cessation are in Table 2.

**Table 1:** Characteristics of patients with neovascular age-related macular degeneration with and without recurrence after cessation of treatment.

	All (*n* = 49)	Non-recurrent (*n* = 24)	Recurrent (*n* = 25)	*P*-value
**Baseline data**				
Age, years	72.8 (8.3)	77.0 (68.0–80.3)	70.0 (65.0–78.0)	0.19*
Sex, n (%) Men	31 (63.3)	13 (54.2)	18 (72.0)	0.244^†^
Anti-VEGF at first, n (%) aflibercept	39 (79.6)	18 (75.0)	21 (84.0)	0.496^†^
**MNV type, *n* (%)**				
Type 1	34 (69.4)	17 (70.8)	17 (68.0)	0.184^†^
(PCV	28 (57.1)	15 (62.5)	13 (52.0) )	
Type 2	10 (20.4)	3 (12.5)	7 (28.0)	
Type 3	5 (10.2)	4 (16.7)	1 (4.0)	
PED at baseline, n (%)	39 (79.6)	20 (83.3)	19 (76.0)	0.725^†^
**Treatment data**				
Treatment period, months	18.8 (12.3–26.2)	20.9 (16.5–28.4)	13.8 (10.6–20.9)	0.008*
Total injections, n	10 (8–12)	11.5 (9.0–13.3)	8.0 (7.0–11.0)	0.004*
Injections for a dry macula in loading phase, n	1 (1–1)	1.0 (1.0–1.3)	1.0 (1.0–2.0)	0.959*
Injections in the 6 months before cessation	3 (2–3)	2 (2–3)	3 (2–3)	0.002*
Injections in the 1 year before cessation	5 (4–6)	5 (4–6)	6 (5–6)	0.119*
Last treatment interval, weeks	13.6 (12–16)	15.4 (13.4–16.0)	12.0 (12.0–14.0)	0.003*
Dry macula period before cessation, months	16.3 (12.2– 21.7)	19.3 (15.8–26.3)	12.8 (9.6–18.8)	0.004*
**OCT characteristic at cessation, *n*, (%)**				
PED				
Serous	0	0	0	1^†^
Fibrovascular	19 (38.8)	11 (45.8)	8 (32.0)	0.387^†^
Atrophy	15 (30.6)	9 (37.5)	6 (24.0)	0.364^†^
**BCVA, logMAR**				
Baseline		0.52 (0.38–0.86)	0.40 (0.30–0.70)	0.227^*^
Last injection		0.26 (0.16–0.82)	0.16 (0.05–0.40)	0.080^*^
Recurrence			0.22 (0.05–0.30)	–
After retreatment			0.16 (0.00–0.40)	–
Last visit		0.30 (0.16–0.52)	0.10 (0.00–0.52)	0.165^*^

Data are presented as median (Interquartile range), unless otherwise indicated. BCVA, best-corrected visual acuity; IRF, intraretinal fluid; logMAR, logarithm of the minimum angle of resolution; MNV, macular neovascularization; OCT, optical coherence tomography; PED, pigment epithelial detachment; SRF, subretinal fluid; VEGF, vascular endothelial growth factor. *Mann–Whitney U test. †A chi-square test.

**Table 2:** Macular findings on OCT determined at recurrence after cessation.

	(*n* = 25)
Hemorrhage	4
Fluid	
SRF + IRF + PED	1
SRF + PED	2
SRF	15
IRF	2
PED	1

IRF, intraretinal fluid; OCT, optical coherence tomography; PED, pigment epithelial detachment; SRF, subretinal fluid;

Approximately 80% of patients received aflibercept from the first injection. Of the 10 eyes that started treatment with ranibizumab, 3 eyes (one in the non-recurrent group and two in the recurrent group) were switched to aflibercept due to inadequate response. Most patients showed a favorable course and the treatment interval was extended smoothly in this cohort. The treatment interval was shortened in four patients (17%) in the non-recurrent group and in three patients (12%) in the recurrent group (*P* = 0.70) before cessation. There were no significant differences in MNV subtypes or optical coherence tomography (OCT) characteristics between the two groups.

**Figure 1:**
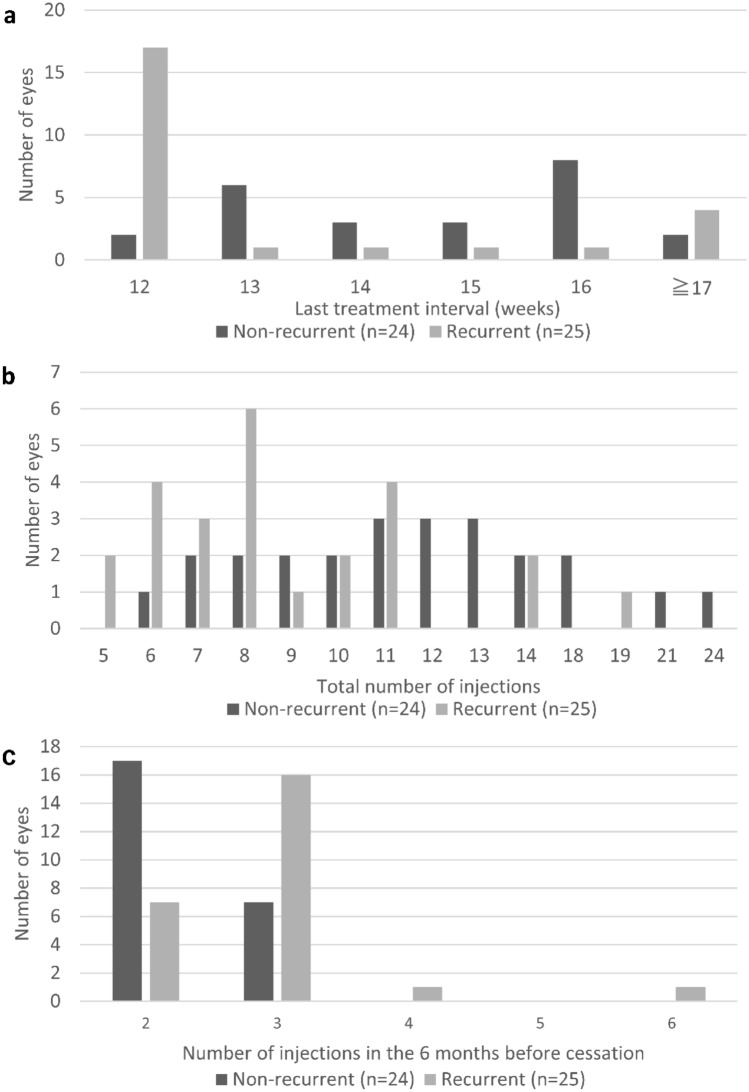
Distribution of (**a**) last treatment interval, (**b**) total number of injections and (**c**) number of injections in the 6 months before cessation in patients with neovascular age-related macular degeneration who stopped treat and extend regimen of anti-vascular endothelial growth factor therapy. Recurrence was frequent in patients with last treatment interval of 12 weeks, total number of injections of less than 12 and number of injections in the 6 months before cessation of 3 or more.

Total treatment period before cessation was longer in the non-recurrent group than the recurrent group (median 20.9 months vs. 13.8 months, *P* = 0.008), and total number of injections was larger in the non-recurrent group (median 11.5 vs. 8.0, *P* = 0.004). The number of injections in 6 months before cessation was significantly larger in recurrent group (median 2 vs. 3, *P* = 0.002). The non-recurrent group also had a longer last treatment interval (median 15.4 weeks versus 12.0 weeks, *P* = 0.003) and a longer dry macula period before the cessation (median 19.3 months vs. 12.8 months, *P* = 0.004) than the recurrent group. Of note, the total treatment period was closely correlated with the total number of injections and the dry period (*R* = 0.96, *p* < 0.001; *R* = 0.91, *p* < 0.001). The distribution of the last treatment interval and the number of injections are shown in Fig. 1. The recurrence rate was 89% in patients with a last treatment interval < 13 weeks.

The best-corrected visual acuity (BCVA) tended to be worse in the non-recurrent group at baseline and cessation. Patients without macular atrophy at cessation had better vision (median 0.16 logarithm of the minimum angle of resolution [logMAR] vs 0.82 logMAR, *P* < 0.01), and all patients with logMAR < 0.1 had no atrophy. Five patients with BCVA worse than 1.0 logMAR at cessation had atrophy at cessation and showed no recurrence. The degree of improvement with treatment was similar between non-recurrent group and recurrent group (median − 1.8 logMAR vs. − 2.3 logMAR, *P* = 0.24).

### Time and proportion of recurrence

**Figure 2:**
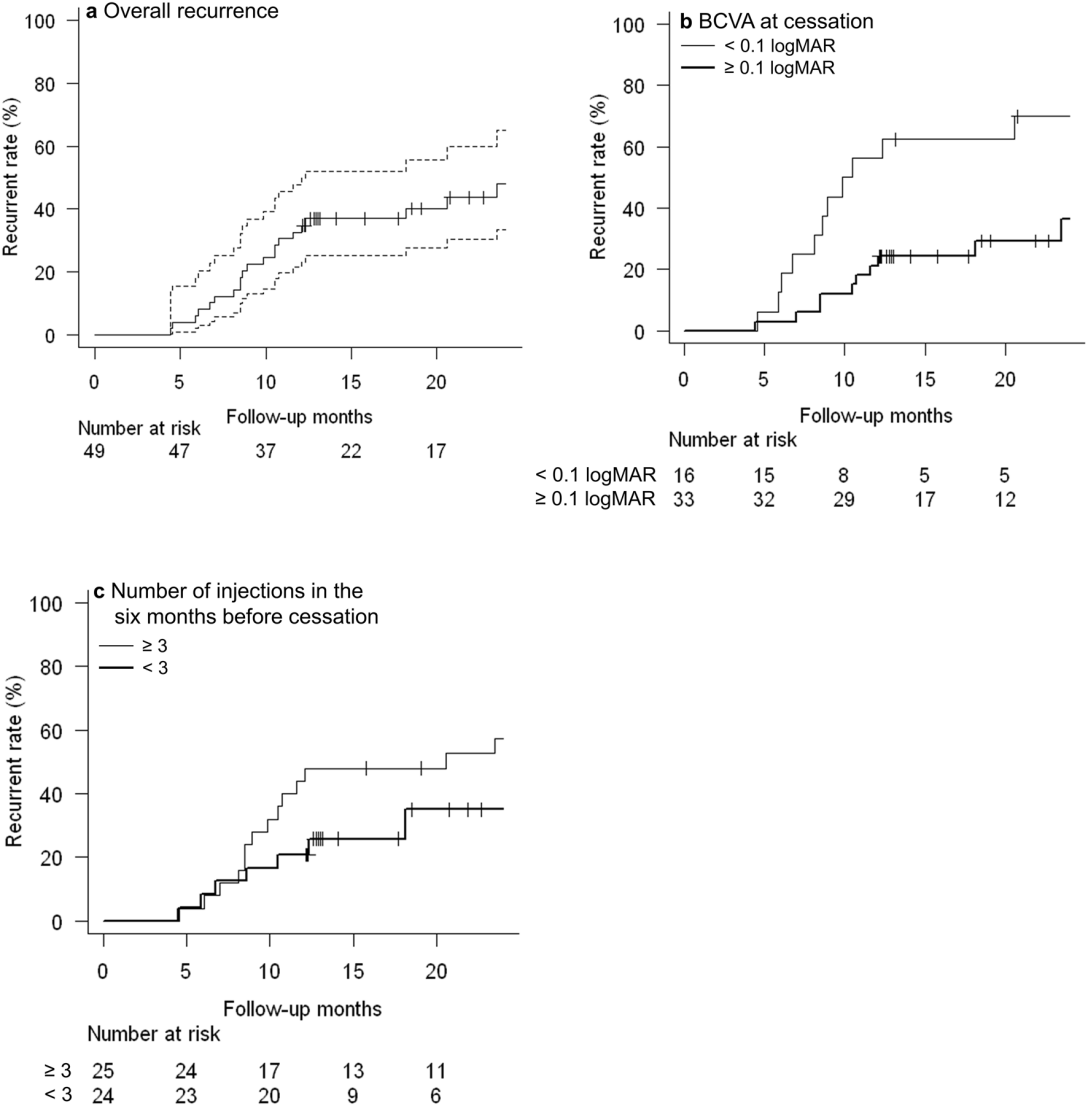
Kaplan–Meier survival curves of recurrence after the cessation of treatment in patients with age-related macular degeneration. (**a**) The estimated recurrence rate was 33% at 12 months and 48% at 24 months, respectively. Patients with (**b**) best corrected visual acuity (BCVA) < 0.1 logMAR at cessation and (**c**) number of injections in the 6 months before cessation ≥ 3 had higher chance of recurrences.

Kaplan–Meier survival curves of the time and proportion of overall recurrence are presented in Fig. 2a. The estimated recurrence rate after treatment discontinuation was 33% (95% confidence interval [CI], 21–48%) after 1 year and 48% (CI, 36–65%) after 2 years from treatment cessation. The median time to recurrence was 30.3 months (95% CI, 12.3–31.7 months).

Cox proportional hazards analysis showed that BCVA at cessation and the number of injections in the 6 months before cessation were associated with the risk of recurrence (Table 3). BCVA < 0.1 logMAR (*P* = 0.02) at cessation, and the number of injections in the 6 months before cessation of ≥ 3 (*P* = 0.073) were associated with recurrence risk. (Fig. 2b, c) These thresholds were determined using receiver operating characteristic (ROC) curves. None of the factors correlated with the time to recurrence.

**Table 3:** Cox proportional hazard analysis of risk factors on recurrence after cessation of treatment in patients with neovascular age-related macular degeneration.

	Hazard ratio	Lower 95% CI	Upper 95% CI	*P* value
BCVA at a last injection	0.135	0.024	0.756	0.024
Number of injections in the 6 months before cessation	2.047	1.252	3.349	0.004

BCVA = best-corrected visual acuity.

### Clinical courses after the recurrences

After recurrence, the patients were re-treated with anti-VEGF agents, photodynamic therapy, and/or retinal photocoagulation. Only two patients (8%) had a visual acuity loss of ≥ 0.2 logMAR due to recurrence. Twelve patients stopped treatment again and showed no recurrence until the last visit. Ten patients continued treatment with TAE or a fixed dosing. One patient dropped out, one was transferred to another clinic, and one died. The mean follow-up period after recurrence was 34.3 ± 14.9 months. There were no significant differences in visual acuity at cessation, at recurrence, and at the last visit (0.16 logMAR, 0.22 logMAR, 0.1 logMAR, respectively) (*P* = 0.84).

### Course of the non-recurrent group

The median follow-up time in the non-recurrent group was 18.8 months (IQR 13.0–24.5 months). The median BCVA at the last visit was 0.3 logMAR and was not significantly different from that in the recurrent group (*P* = 0.37). Four patients (17%) had a visual loss of ≥ 0.2 logMAR from cessation to the last visit.

## Discussion

We investigated the incidence and risk factors for recurrence after treatment cessation. It was found that 51% of patients with nAMD experienced recurrence at a median of 10.5 months after cessation of TAE. The non-recurrent group had a longer treatment period, longer last treatment interval, longer dry period, and a larger number of injections before cessation. BCVA at cessation and the number of injections in 6 months before cessation were associated with the risk of recurrence.

There are several reports on the cessation of the TAE regimen and subsequent recurrences (Table 4)^13–21^. Adrean et al.^13^ proposed Treat, Extend, and Stop protocol, in which continuous treatment is posed after two consecutive injections by extending the treatment interval to 12 weeks with careful TAE. The recurrence rate was 16% in 1 year. Munk et al.^14^ stopped treatment after three 16-week-interval injections and reported a recurrence rate of 15% within 9 months. Other studies showed a 38–53% recurrence rate in 1 year^15,18–21^.

**Table 4:** Summary of previous reports on recurrence after cessation of treat and extend regimen in patients with neovascular age-related macular degeneration.

	Year	Study design	Past treatment	Cessation cases, n	Treatment period, mean (SD)	Total injection, n mean (SD)	Last treatment interval	12 months recurrence rate	Overall recurrence rate, Mean time to recurrence	Risk factors of recurrence
Adrean et al^13^	2018	Retro	–	143	–	22 (range, 7–48)	12 weeks × 2	16%	29%, 14 months	None
Munk et al^14^	2018	Retro	Some had PRN	100	4.5 ± 2.5 years	23.7 (14.7)	16 weeks × 3	–	15%, 41 ± 7 weeks	High risk: Vitreomacular adhesion Low risk: rapid responder
Nguyen et al^15^	2019	Pro	Any treatment was allowed	434	median 23 (IQR, 63–138) months	median 10 (IQR, 7–14)	median 11 weeks (IQR 9–14)	41%	63%, median 16.8 (IQR,13.3–20.3) months	Low risk: low visual acuity at cessation and long treatment period
Obana et al^18^	2021	Retro	96% were naïve	88	–	8.5 (3.1)	16 weeks × 2	–	32%, 13.2 ± 10.1 months	none
Aslani et al^19^	2021	Pro	–	102	–	10.6 (4.4)	12 weeks	53%	53%, 6.7 ± 2.2 months	High risk: PED at baseline
Garweg et al^20^	2022	Retro	Naïve	54	22.3 ± 10.1 months	11.0 (4.8)	14 weeks	40%	61%, 9.2 ± 6.7 months	none
Matsubara et al^21^	2022	Retro	PRN or fixed dose were also allowed	34	median 34 (IQR, 24–59) months	median14 (IQR, 12–22)	12–16 weeks (80% were 12 weeks)	38%	50%, median 10 (IQR 6–12) months	None
This study		Retro	Naïve	49	median 19 (IQR, 12–26) months	median 10 (IQR 8–12)	≥ 12 weeks	33%	51%, median 10.5 (IQR 8.1–18.2) months	Low risk: low visual acuity at cessation and large number of injections

IQR, interquartile range; PED, pigment epithelial detachment; PRN, pro re nata; SD, standard deviation.

Among the previously reported predictive factors for recurrence, low visual acuity at treatment cessation was also associated with fewer recurrences in our study. The association between poor visual acuity and fewer recurrences was explained, such that, eyes with reduced visual acuity also had retinal atrophy, which had lower risk of recurrence because the activity of their MNV is already withered. In fact, all five patients with BCVA worse than 1.0 logMAR at cessation and who did not show recurrence, had macular atrophy. Additionally, good visual acuity (VA) was a risk factor for recurrence in the present study. We considered that patients with good VA and good response to treatment tend to stop treatment early and the insufficient number of injections lead recurrence. However, this was not the case because there was no correlation between VA and the treatment period or number of injections. Atrophy may be one reason; eyes with good vision generally have no atrophy and showed more recurrences. However, it remains still unclear why good VA is associated with a higher incidence of recurrence.

In the present study, 80% of the patients in the recurrent group had a last treatment interval of 13 weeks or less, suggesting that the last treatment interval of 12 weeks was too short. Meanwhile, no recurrence within 12–16 weeks treatment interval is a common criterion for treatment cessation, and treatment interval seemed to have little effect on the recurrence rate in previous studies (Table 4). In addition, the median time prior to recurrence in the present study was 8.5 months, and the shortest case was 4.4 months. Thus, the treatment interval could be safely extended to 16 weeks in cases with a last treatment interval of 13 weeks or less. It is unreasonable to consider that most of these subsequent recurrences were prevented if a few additional injections were administered in these cases. In fact, multivariate analysis suggests that the last treatment interval is a confounding factor but is not an independent factor for the risk of recurrence.

The large number of injections in the 6 months before cessation was a significant risk factor. A larger number of injections in the 6 months before cessation means that treatment interval was extended at a high pace and treatment was discontinued after a short observation. The result indicates that even in patients with a long last treatment interval, a rapid interval extension would not sufficiently suppress MNV, leading to recurrence. This may also explain why the last treatment interval was not associated with a significant risk of recurrence. Further studies are needed to determine the relationship between the last treatment interval, number of injections and risk of recurrence.

There is a trade-off between the number of injections and visual outcome. Continuous treatment is a burden but is associated with a lower risk of recurrence. In contrast, discontinuation of treatment may reduce the burden but is associated with the risk of recurrence. When considering the risk of recurrence, the outcome of the recurrence management is important. Previous studies^13,19^ reported that recurrence after treatment cessation can generally be controlled well with maintained vision, which is consistent with the present study. Among the 25 recurrences, only two patients lost two lines of vision despite resumed treatment. The MNV activity may be lower in patients who discontinue treatment, and the recurrence may be less severe in the overall nAMD cohort. Treatment cessation can be relatively safe in these patients if continuous monitoring is performed.

The present study has several limitations, including its retrospective design and relatively small sample size. We did not define a pachychoroid. The decision to discontinue treatment was at the physician’s discretion. The studied patients may have been biased toward those with a good course and do not reflect the total AMD cohort. Although we investigated the last treatment interval, it was between 12 and 16 weeks in most patients. Further studies with larger cohorts are needed to establish optimal indications for the cessation of treatment.

Here, we report the incidence and clinical course of recurrence after cessation of the TAE regimen in patients with nAMD. One-third of the patients experienced recurrence within 1 year after cessation of treatment, but overall, recurrence was observed in half of the patients thereafter. Recurrence was more frequent in patients with better vision, larger injections in the 6 months before cessation, and less frequent in patients with poor vision. The final treatment interval was not significantly associated with recurrence. Even after recurrence, vision was maintained even after retreatment.

## Methods

### Study design

This was a multicenter, retrospective study. The study design was approved by the Institutional Review Board of Nagasaki University and the Japanese Red Cross Nagasaki Genbaku Hospital, and all study conduct adhered to the tenets of the Declaration of Helsinki. The requirement for written informed consent was waived by the ethics committee, given the retrospective nature of the study. Instead, patients were allowed “opt-out” consent.

### Patient selection and intervention

Patients with treatment-naïve nAMD receiving intravitreal injections of ranibizumab or aflibercept under a TAE regimen between January 2015 and December 2021 were identified in our database at the Department of Ophthalmology at Nagasaki University Hospital and Japanese Red Cross Nagasaki Genbaku Hospital.

We included patients who stopped continuous treatment after the treatment interval was extended to ≥ 12 weeks. Participants were excluded from the study if they were followed up for less than 12 months after the last injection or had other macular or optic nerve diseases. If both eyes met the inclusion criteria, the right eye was selected and studied.

At baseline, participants underwent a comprehensive ophthalmic examination. BCVA was measured using LogMAR. The diagnosis of AMD subtype, e.g., type 1 MNV with or without polypoidal lesions, type 2 MNV, type 3 MNV, was made based on fundoscopy, fundus photography, retinal angiography (fluorescein and indocyanine green), spectral-domain OCT (SD-OCT, Spectralis HRA + OCT instrument, Heidelberg Engineering GmbH, Heidelberg, Germany), and OCT angiography (Angio Vue, OptoVue Inc., Freemont, CA, USA). Retinal angiography was not performed when the patient had a history of drug allergy, active asthma, or renal dysfunction.

All patients initially received three monthly anti-VEGF injections. Thereafter, treatment intervals were extended or shortened at the discretion of the treating physician depending on the retinal exudative change: new retinal hemorrhage, presence of intraretinal and/or subretinal fluid, or enlargement of pigment epithelial detachment (PED) on SD-OCT. In principle, the treatment interval was extended by one or two weeks when there was no sign of exudation and shortened by two weeks or more when exudative changes were noted. When the treatment interval was extended to longer than eight weeks, patients were instructed to visit local ophthalmologists in between. Only patients determined to have no intra or sub- retinal fluid in the macula and stopped treatment were included in the study. Recurrence was defined as the presence of any intra or sub-retinal fluid after cessation. In cases of recurrence, anti-VEGF injections were resumed. The date of recurrence was determined based on the patient’s medical history. When the patient was asymptomatic, we assumed that recurrence developed at the time of when the fluid was confirmed on OCT images.

### Outcomes

The primary outcome measure was the incidence of post-cessation recurrence. Secondary outcome measures included the effect of BCVA, total treatment period, number of injections, last treatment interval, dry period, and OCT parameters before the cessation of the risk and timing of recurrence. “Dry period” was defined as a non-recurrent period without retinal exudative changes in the macula.

### Statistical Analysis

All statistical analyses were performed using EZR (Saitama Medical Center, Jichi Medical University, Saitama, Japan), a graphical user interface for R (The R Foundation for Statistical Computing, Vienna, Austria)^22^. More precisely, it is a modified version of the R commander designed to add statistical functions frequently used in biostatistics. Significance tests for continuous variables were performed using the Mann–Whitney U test. Wilcoxon’s signed-rank test was used to compare changes in BCVA. The chi-square test was used to analyze nominal variables. The proportion and time to recurrence were analyzed using Kaplan–Meier survival analysis. The correlation between various parameters was tested using Spearman's rank correlation coefficient. Cox proportional hazard regression analysis was performed to estimate the hazard ratio for recurrences using univariate and multivariate analyses, number of injections in the 6 months before cessation, and BCVA at cessation. The selection of a priori variables was based on the results of univariate analyses and clinical perspectives. Statistical significance was set at *p* < 0.05.

## Data Availability

All the relevant data are presented in the manuscript. The datasets generated and analyzed during the current study are available from the corresponding author upon reasonable request.
